# Fragment Screening of Human Aquaporin 1

**DOI:** 10.3390/ijms17040449

**Published:** 2016-03-25

**Authors:** Janet To, Jaume Torres

**Affiliations:** School of Biological Sciences, Nanyang Technological University, 60 Nanyang Drive, Singapore 637551, Singapore; yyto1@ntu.edu.sg

**Keywords:** human aquaporin 1, fragment based drug discovery, surface plasmon resonance, thermal shift, membrane protein

## Abstract

Aquaporins (AQPs) are membrane proteins that enable water transport across cellular plasma membranes in response to osmotic gradients. Phenotypic analyses have revealed important physiological roles for AQPs, and the potential for AQP water channel modulators in various disease states has been proposed. For example, AQP1 is overexpressed in tumor microvessels, and this correlates with higher metastatic potential and aggressiveness of the malignancy. Chemical modulators would help in identifying the precise contribution of water channel activity in these disease states. These inhibitors would also be important therapeutically, e.g., in anti-cancer treatment. This perceived importance contrasts with the lack of success of high-throughput screens (HTS) to identify effective and specific inhibitors of aquaporins. In this paper, we have screened a library of 1500 “fragments”, *i.e.*, smaller than molecules used in HTS, against human aquaporin (hAQP1) using a thermal shift assay and surface plasmon resonance. Although these fragments may not inhibit their protein target, they bound to and stabilized hAQP1 (sub mM binding affinities (*K*_D_), with an temperature of aggregation shift Δ*T*_agg_ of +4 to +50 °C) in a concentration-dependent fashion. Chemically expanded versions of these fragments should follow the determination of their binding site on the aquaporin surface.

## 1. Introduction

Aquaporins (AQPs) are membrane proteins that permeabilize cellular membranes to water [1,2,3]. In humans, 13 AQPs have been identified [4], but only AQP0, AQP1, AQP2, AQP4, AQP5 and AQP8 are water-specific (*i.e.*, “orthodox” aquaporins), while the rest are also permeable to glycerol (referred to as “aquaglyceroporins”), although many other substrates for transport across membranes have been reported [5,6,7,8,9,10,11,12,13,14]. The structure of AQPs is well conserved across species; each AQP monomer has six α–helical transmembrane (TM) domains, with two “loops” that enter the membrane and face each other through a conserved NPA motif, and where both N- and C-terminal tails are cytoplasmically oriented [15,16,17,18,19,20,21]. AQPs form homotetramers, and each monomer in the tetramer functions independently as a water channel [22].

Over the last 18 years, phenotypic analyses have identified physiological and pathological implications for AQP function (reviewed in [23]), e.g., in brain swelling or glandular fluid secretion. The potential for AQP water channel inhibitors in cerebral edema [24,25,26,27,28], water retention [9,29,30,31,32], or regulation of eye intraocular pressure (IOP) [33] associated with glaucoma [34], and others, has also been discussed [35,36,37,38]. Recently, it has been shown that AQPs are new players in angiogenesis and cancer biology [39]. Indeed, a growing number of reports over the last 10 years hypothesize that it is the increased water flow through AQP channels, induced by local osmotic gradients, that is responsible for the formation of membrane protrusions during cell migration [37,39,40], a general phenomenon observed for several cell types and different AQPs, e.g., AQP4 [41], AQP1 [42], AQP3 [43,44] or AQP9 [45,46,47,48]. Several AQPs are upregulated in many types of cancer [49], and expression correlates with a higher tumor grade [50].

Specifically for AQP1, its expression in liver endothelial cells contributes to amoeboid invasion in liver angiogenesis and cirrhosis [51]. AQP1 is abnormally expressed in tumor endothelial cells [52,53], whereas AQP1 deletion in knockout mice greatly impaired tumor growth, angiogenesis and cell migration [52]. AQP1-expressing tumors have more metastatic potential *versus* AQP1-null tumors [54]. siRNA against AQP1 reversed tumor growth in a murine model of melanoma [55]. Despite these supporting data, the precise role of AQP1 is not yet clear, as interactions of AQPs with the cytoskeleton or other proteins [56,57] may be at least as important as increased water flow in the formation of cell protrusions. The precise role of aquaporins in cell migration is still elusive without the availability of specific, potent and non-toxic water channel inhibitors of AQPs. In addition, hAQP1 inhibitors would have obvious potential therapeutic implications to complement current anti-cancer treatments, e.g., anti-VEGF therapy, where resistance to anti-VEGF treatment has been described [58,59,60,61].

Despite the important therapeutic implications, to our knowledge, not a single organic molecule has been reported in the literature to be effective in the inhibition of AQP1 using standard assays, e.g., red blood cells, transepithelial assays or AQP1-proteoliposomes, even less at nM concentrations. For example, current reported AQP1 or AQP4 inhibitors are sulfhydryl-reactive mercurials, e.g., HgCl_2_ [51,62,63], heavy metals [63,64,65], quaternary ammonium salts, e.g., tetraethylammonium (TEA+) [66,67,68], inorganic salts [69], loop diuretic co-transporter blockers [70], pan-inhibitor of carbonic anhydrase acetazolamide [71,72,73], or TGN-020 (2-(nicotinamoyl)-1,3,4-thiadiazole) [74]. However, many of these compounds are either toxic, not potent, lack specificity, are unsuitable for drug discovery or their inhibitory effect has been heavily disputed [75,76].

Conventional high-throughput screening (HTS) campaigns to search for inhibitors have not been successful. In a reported 100,000 compound screen by the Verkman lab, no significant water channel inhibitors were found [39]. Compared to other common membrane proteins, such as enzymes, whose active sites can act as a binding pocket for drug modulators, the natural architecture of the aquaporin water channel is conceptualized to have a “slippery” hourglass shape that has a narrow pore diameter, restricting the anchoring of drug modulators. Despite this, some aquaporins have been shown to display gating mechanisms that could be druggable, e.g., spinach plasma membrane aquaporin SoPIP2;1 [77], where dephosphorylation of two serine residues under drought stress can trigger occlusion of the pore. In addition, AQP4 water permeability is regulated in a similar way, by changing the phosphorylation state of Ser-111 and Ser-180, on B and D loops [78,79,80], although these gating mechanisms have been disputed [81,82,83]. Finally, for AQP0, calmodulin (CaM) binds to its cytoplasmic C-terminal domain in a Ca^2+^-dependent manner [84], which results in the closure of its cytoplasmic gate and inhibition of water permeability [85].

An alternative to HTS is fragment-based drug discovery (FBDD) [86,87], where highly sensitive and robust biophysical techniques [88] are required for the identification of small molecule “fragments” (~120 to 250 Da), which generally bind weakly to their targets, with affinities typically in the range of 10 µM–1 mM. Although these “fragments” do not necessarily inhibit their protein target, they may constitute good starting points that can be derivatized or linked to other fragments to produce a suitable inhibitor. In addition, owing to their structural simplicity, fragments frequently participate in better-quality interactions as compared to drug-sized compounds [89]. Fragments have lead-likeness properties [90] so that properties can be easily optimized to improve drug potency, are more efficient in exploring the binding sites of proteins [86,89] and have higher binding energy per unit molecular mass than larger compounds. Finally, libraries are vastly smaller than those used in HTS campaigns. The selection of fragments is usually followed by structural determination of binding sites and the mode of action. Surface plasmon resonance (SPR) produces quantitative binding information to rank top binders by affinity [89] with a consumption of protein at least 10- to 100-fold lower than for other biochemical and biophysical screening methods for fragments [91].

During the past five years, attempts to screen fragment ligands for integral membrane proteins using biophysical methods, e.g., SPR or target immobilized NMR screening (TINS), have been described. For example, for *E. coli* DsbB [92], thermostabilized (StaR) G protein-coupled receptors (GPCRs) [93,94], or fatty acid amide hydrolase (FAAH) [95] using ^19^F NMR (*n*-fluorine atoms for biochemical screening, *n*-FABS). However, these applications are still scarce due to the difficulty in the handling of membrane proteins, which has required in many cases the use of fusion constructs or thermostabilized mutants.

The present paper uses the native unmodified form of hAQP1 and is the first reported application of biophysical fragment screening in aquaporins of any kind. We have used SPR and thermal shift [96] assays. The latter was performed as a differential static light scattering (DSLS) mode, which is suitable for studying membrane proteins in detergent micelles [97,98].

## 2. Results

### 2.1. Expression and Purification of Human Aquaporin 1 (hAQP1) from Insect Cells

**Figure 1:**
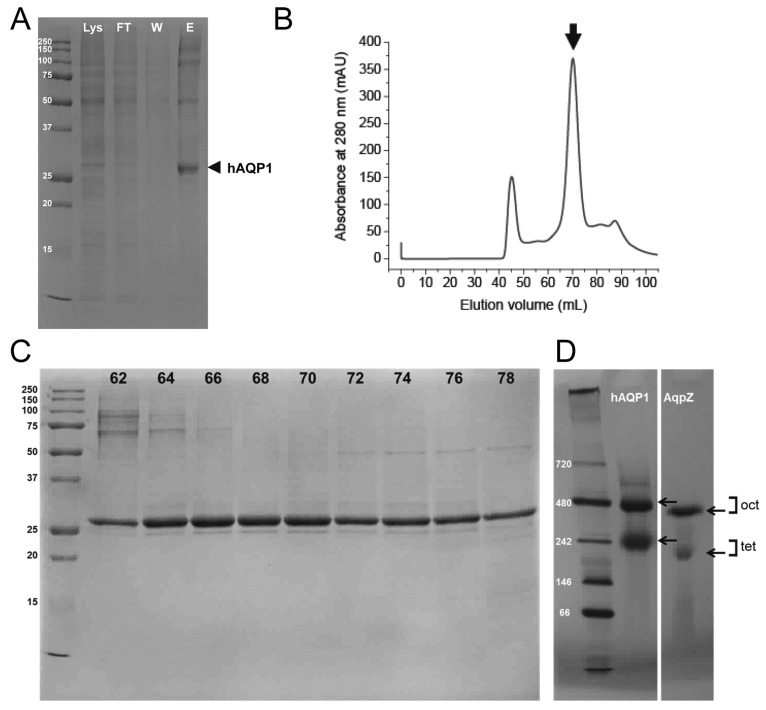
Purification and oligomeric status of human aquaporin 1 (hAQP1). (**A**) fraction analysis of Ni-NTA purification profile in SDS-PAGE of hAQP1 from Sf9 cells: Lys, whole cell lysate; FT, flow-through; W, wash; E, eluent. The arrow points to the purified hAQP1 monomer at ~31 kDa; (**B**) gel filtration profile of hAQP1 purification. The major peak eluted at 70 mL (arrow) and corresponds to the tetrameric hAQP1 (~124 kDa). The minor peak at 45 mL (void volume, *V*_0_) contained aggregates of hAQP1; (**C**) SDS-PAGE of the fractions from 62 to 78 mL eluted in the gel filtration chromatography. The protein amount loaded per lane was 5 µg; and (**D**) Blue Native PAGE (BN-PAGE) of purified hAQP1 in 100 mM *n*-octyl-β-d-glucopyranoside (OG) and purified AqpZ as an internal control. The hAQP1 band at ~242 kDa corresponds to tetramers (tet, expected 124 kDa) and band at ~480 kDa corresponds to its octamer (oct, expected 248 kDa).

The recombinant hAQP1 with an N-terminal histidine tag and tobacco etch virus (TEV) protease cleavage site (6His-TEV-hAQP1) was present in the soluble fraction of the cell lysate, thereby permitting direct protein extraction in the presence of detergent *n*-octyl-β-d-glucopyranoside (OG). Typically, 16–18 g of cell pellets can be obtained per liter culture. Protein purification was completed using nickel-affinity chromatography followed by gel filtration using fast protein liquid chromatography (FPLC). The fractions from the nickel-affinity column were analyzed by SDS-PAGE (Figure 1A). The recombinant hAQP1 (monomeric size of 31,136 Da) migrated as a monomer in SDS, although high molecular weight oligomers or contaminants were also present. Eluted fractions were concentrated and subjected to further purification by gel filtration. From the chromatogram profile obtained, hAQP1 eluted as a single major peak at 70 mL (Figure 1B, arrow), which represents the tetrameric state of the hAQP1, *i.e.*, a molecular weight of ~124 kDa. Therefore, the recombinant hAQP1 obtained is monodisperse and mainly exists as a tetramer in presence of OG. A minor peak in the chromatogram, representing higher molecular weight aggregates of the protein, eluted at the void volume (*V*_0_) of the gel filtration column, around 45 mL. All the fractions collected from the major eluted peak contained hAQP1 (Figure 1C). In this SDS-PAGE gel, hAQP1 migrated as a monomer, with estimated sample purity of ~95%. Typically, a yield of 5–6 mg of purified hAQP1 could be obtained per liter of culture.

The sample in OG was analyzed by blue-native polyacrylamide gel electrophoresis (BN-PAGE) (Figure 1D) [99]. In this case, interference of the dye and the presence of detergent contribute to the overall size of the protein complex and can result in an additional mass with a factor of ~1.8 [100]. For comparison, we included as internal membrane protein standard purified *E. coli* aquaporin Z (AqpZ), shown to form tetramers in OG with apparent molecular weight of ~170 kDa (expected 100 kDa) [101]. Based on the above, the hAQP1 band at ~242 kDa should correspond to its tetramer (expected 124 kDa). The hAQP1 band at ~480 kDa is assigned to its octamer (expected 248 kDa), since an octamer is also observed for AqpZ at ~400 kDa.

### 2.2. Water Permeability of Purified hAQP1

**Figure 2:**
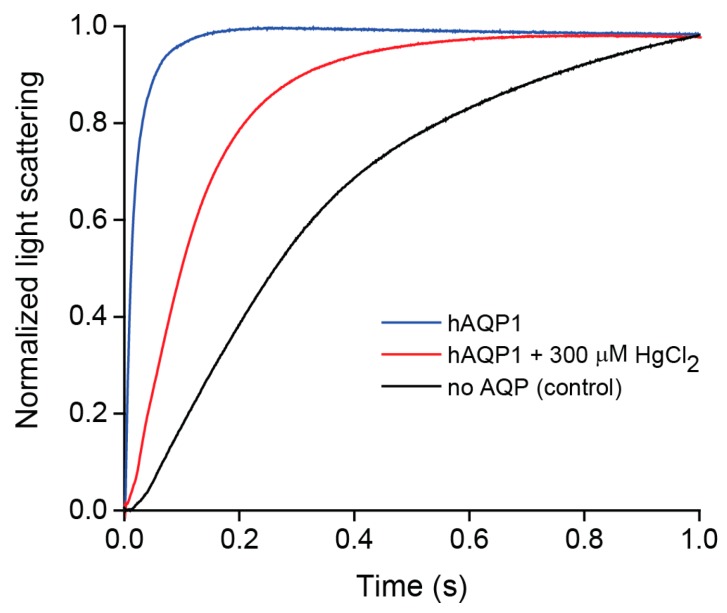
hAQP1 water permeability functional assay. Normalized light scattering intensity changes of hAQP1—*E. coli* lipid proteoliposomes (**blue** trace) compared with no-AQP liposome control (**black** trace) in response to an osmotic shock, and inhibition of hAQP1 proteoliposomes by mercuric chloride (**red** trace). The average fitted rate constants (*k*) (means ± S.E., *n* = 6) measured at 23 °C for hAQP1-proteoliposomes, proteoliposomes in presence of 300 µM HgCl_2_ and no-AQP liposomes were 109.4 (±1.9), 8.56 (±0.03) and 2.27 (±0.04) s^−1^, respectively.

In response to an osmotic shock, the calculated osmotic water permeability coefficient (*P_f_*) of the hAQP1-proteoliposomes (*P_f_* = 0.053 cm/s) was almost 40 times greater than that of the control liposomes (*P_f_* = 0.0015 cm/s) (Figure 2). This enhancement is comparable to that described in the literature [102], demonstrating that hAQP1 purified from insect cells is functional. hAQP1 was also sensitive to inhibition by mercuric chloride, a known potent inhibitor of AQP1 [63]. Osmotic water permeability of hAQP1 proteoliposomes pre-incubated with 300 µM HgCl_2_ was reduced by almost 15 times (*P_f_* = 0.0041 cm/s) (Figure 2). A similar value has been observed in the literature [75], supporting that the hAQP1 produced here is active and can be inhibited.

### 2.3. Hit Binding Measured by Surface Plasmon Resonance (SPR)

Once validated the purity and activity of the recombinant hAQP1 obtained from the insect cell expression, the fragment library was screened against hAQP1 using SPR. As described in the Methods section, hAQP1 was immobilized by amine coupling to the carboxymethylated dextran matrix, thereby enabling the protein to move freely [103]. The SPR response depends on the change in mass concentration on surface of the sensor. The latter, in turn, depends on the molecular weight of the analyte in relation to the number of ligands on the sensor surface. *R*_max_ describes the maximal binding capacity of a captured ligand (hAQP1) for the analyte (fragment in the library) in response units, and the theoretical *R*_max_ can be calculated according to:

*R*_max_ = *M*W_analyte_/*M*W_ligand_ × *R*_ligand_ × *V*_ligand_ (1)

where *M*W_analyte_ and *M*W_ligand_ are the molecular weights of the analyte (fragments) and ligand (hAQP1) molecules, respectively; *R*_ligand_ is the response obtained from hAQP1; and *V*_ligand_ is the valency of hAQP1/proposed stoichiometry of the interaction [104]. To generate good and reliable SPR data for studies of protein:small molecules interactions, the *R*_max_ should ideally be ~25 response units (RU), assuming 100% protein biological activity and 1:1 binding mode. Taking a 120-Da fragment as the smallest analyte in this experiment, the theoretical RU of hAQP1 to be immobilized is approximately 6500 RU (calculated using the above equation: 25 RU = (120/31,136) × *R*_ligand_ × 1). However, since it is unlikely that the full protein surface will be active, we immobilized the protein in excess, to 12,000 RU, to ensure that we detect meaningful binding signals. SPR requires very high coupling densities of the target, typically 5000 to 10,000 RU [103].

**Figure 3:**
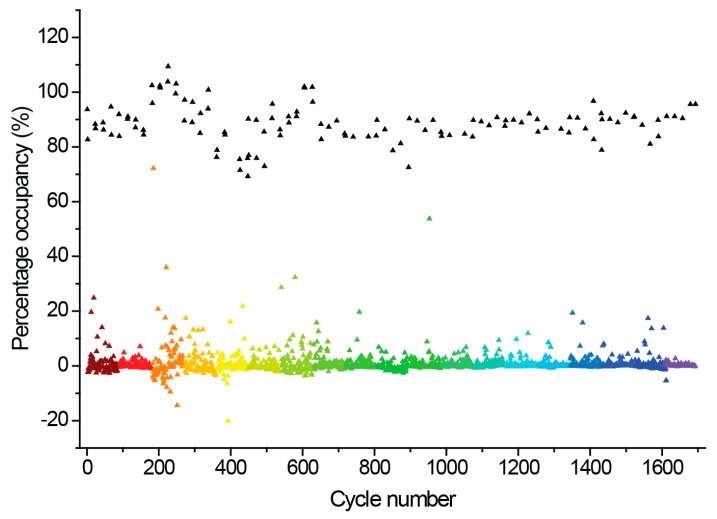
Fragment screening against hAQP1 protein using Surface plasmon resonance (SPR). Screening data adjusted from response unit to percentage surface occupancy, based on equilibrium binding response values (*R*_eq_) obtained from the Biacore T-200 instrument for the 1500-fragment library. Each cycle number represents a different fragment. Each color group represents individual screening plates (total of 19). Screening proceeded for 15 days, and data obtained per screening plate was adjusted so that the baseline scattered evenly around zero percent occupancy. The relative binding occupancy of the positive control, NSC624169, injected at 200 µM every 20 cycles, is also shown (**black** triangles).

In our experiment, the hAQP1 protein immobilized onto the SPR biosensor chip yielded 9000 RU. During the course of screening, control injections of two positive control binders of hAQP1 [97], NSC624169 (*M*W*:* 357) and NSC226080 (*M*W: 914) were conducted at regular intervals (approximately once every 20 compounds, *i.e.*, the start, mid and end cycles relative to each assay plate). This was done to monitor possible surface deterioration and subsequent non-specific binding, which may result in false positives. These positive controls have molecular weights comparable to the average molecular weight of the fragments to be screened, and also gave reproducible binding affinities (*K*_D_) values. The percentage occupancy of each fragment to the immobilized protein was plotted against the injection cycle number (Figure 3).

**Figure 4:**
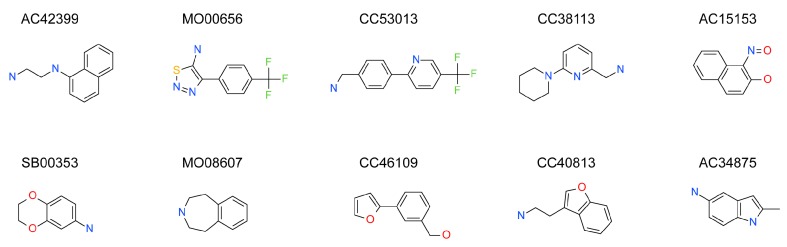
Top 10 fragment binders against hAQP1 discovered using SPR screening.

**Figure 5:**
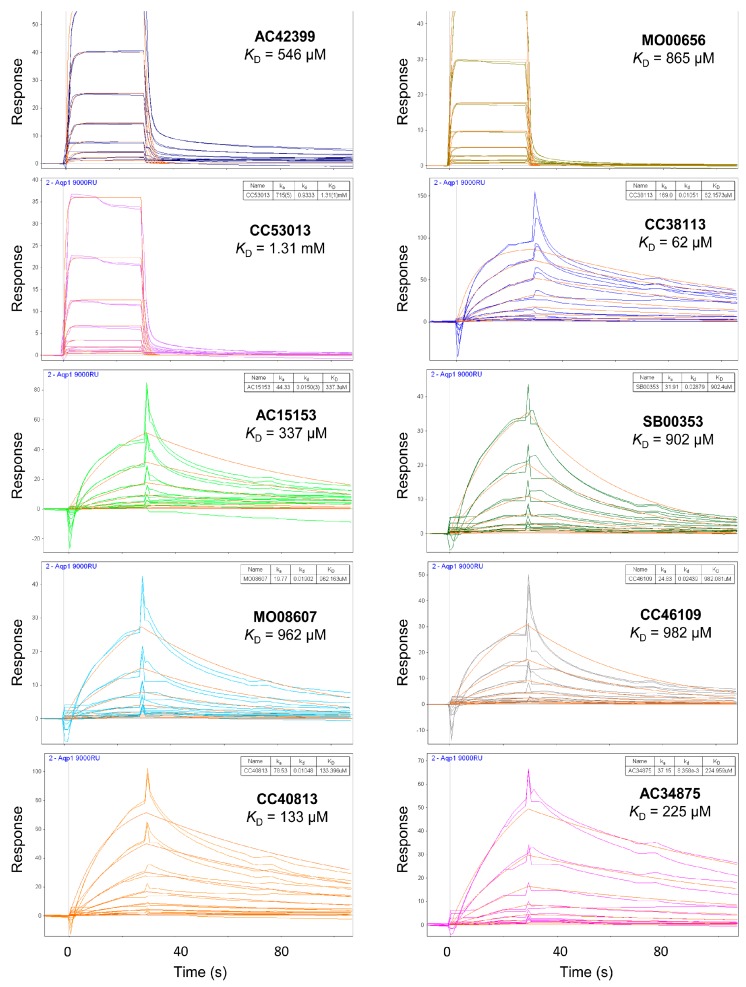
SPR sensorgrams of the top 10 hit fragments against hAQP1 shown in Figure 4. The *y*-axis represents response unit (RU) and the *x*-axis represents time (s). Each fragment was tested in a 2-fold dilution from 6.25 to 800 µM. The steady-state signals plotted as a function of concentration was globally fitted to a typical 1:1 steady-state binding model, giving the binding affinities (*K*_D_) based on 50% occupancy. Experimental data has a different color for each sample, whereas fitted curve is shown in orange.

The data in Figure 3 was used to identify top fragments with good and clean binding to hAQP1. These primary hits were confirmed using secondary SPR experiments in order to eliminate false positives [103]. A total of 22 compounds were chosen for a second stage dose-response assay to be performed at 25, 50, 100 and 200 µM. This secondary assay identified 10 binders (Figure 4), which were further assayed in a concentration range from 6.25 to 800 µM in 2-fold dilutions to study their binding kinetics and *K*_D_ values (Figure 5).

The *K*_D_ values were in the range of high micromolar to low millimolar affinity, which is typical of small molecule fragments. AC42399, MO00656 and CC53013 exhibited stoichiometric binding to the target (Figure 5), with concentration-dependent binding. The traces were fitted to a one-site binding model and were characterized by a fast-on, fast-off kinetics. These are well-behaved fragments that are good candidates for lead generation [103]. In particular, both MO00656 and CC53013 have a trifluoromethyl functional group (Figure 4), suggesting that this chemical moiety may be beneficial for the interaction with hAQP1. For the rest of the fragments, binding was generally observed as a slow rise to equilibrium in most cases and a slow decay back to the baseline. However, in some cases, results were compatible with concentration-dependent aggregation. For example, for CC40813 and AC34875, binding did not achieve saturation equilibrium at higher concentrations, and dissociation was not complete at the end of the run (Figure 5).

### 2.4. Hit Confirmation Using Thermal Aggregation Shift Assay

**Figure 6:**
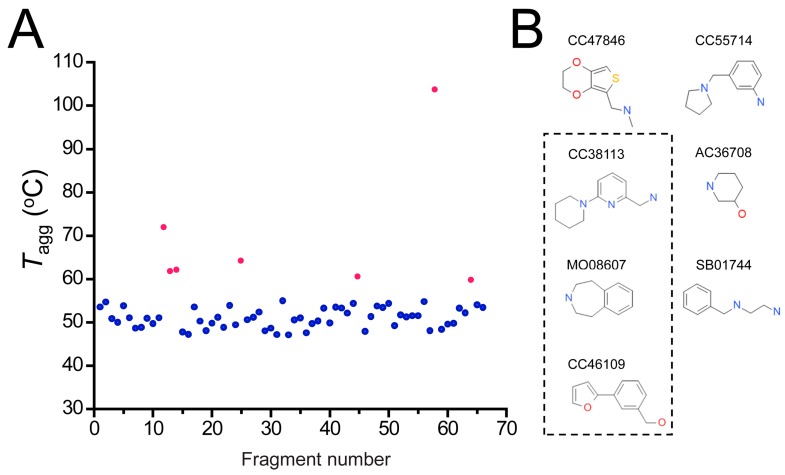
*T*_agg_ of hAQP1 in the presence of hit fragments. (**A**) scatterplot of *T*_agg_ values of hAQP1 in the presence of each of the 66 top fragment binders identified from SPR assay, screened here at 1 mM by differential static light scattering. Each point is an average of three independent measurements. Fragments which stabilized hAQP1 by 1 °C or more are indicated as red dots, whereas the rest are shown in blue; (**B**) structures of the fragments identified in (**A**). Compounds also identified as the top 10 binders by SPR appear inside a box.

In a complementary strategy, hits were tested for their effects on the thermal stability of hAQP1 using differential static light scattering (DSLS), which measures the aggregation of the protein in response to increasing temperature. Since this assay can be performed in a 384-well plate format, the 66 top binders identified from SPR experiments were tested. As the fragments are expected to bind with weak affinity to the target protein, the primary screening concentration was set to 1 mM, and fragments that led to an increase of the aggregation temperature (*T*_agg_) of hAQP1 of 1 °C or more were considered for further examination. Seven fragments improved the thermostability of hAQP1 (Figure 6A, red dots), and are shown in Figure 6B. Out of these seven hits, CC38113, MO08607 and CC46109 were found to overlap with the top 10 hits in the SPR experiment (Figure 6B, boxed).

**Figure 7:**
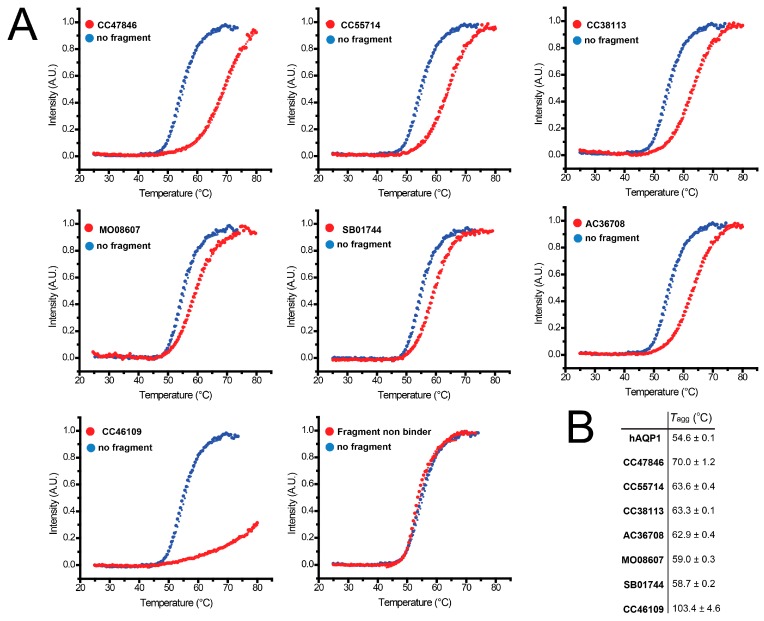
Thermal denaturation curves of hAQP1. (**A**) assays performed in the presence of 1 mM of each of the seven stabilizing hit fragments shown in (**B**). Light scattering intensities are plotted as a function of temperature and fitted to the Boltzmann equation by non-linear regression to obtain the temperature of aggregation, *T*_agg_. Each curve is a representative of three independent experiments with similar results; (**B**) average *T*_agg_ values of purified hAQP1 in the absence and presence of hit fragments.

The thermal denaturation curves in presence of these seven fragments (Figure 7A) show that they have a stabilizing effect, *i.e.*, a positive shift in the *T*_agg_ of hAQP1. A non-binder fragment included as a negative control, TL00706, did not have a significant effect on hAQP1 thermal stability. One of the fragments, CC46109, increased the *T*_agg_ of the protein by ~50 °C when tested at 1 mM (Figure 7B). However, the increase in *T*_agg_ in this case is an estimate, since the protein denaturation curve did not achieve saturation in the temperature range of the experiment.

**Figure 8:**
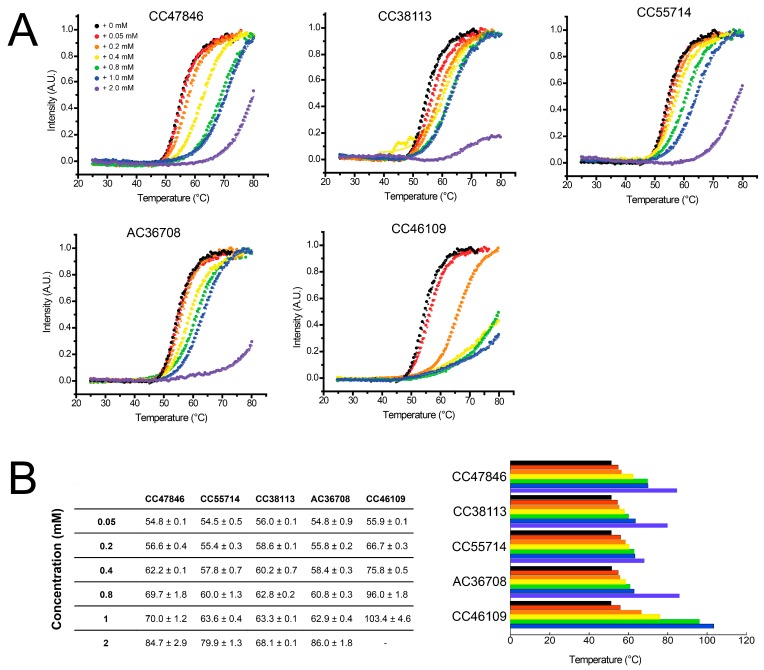
Dose-dependency of hit fragments on *T*_agg_ of hAQP1. (**A**) thermal denaturation curves of hAQP1 in the presence of increasing concentrations of each of the five selected hit fragments. Light scattering intensities are plotted as a function of temperature and fitted to the Boltzmann equation by non-linear regression to obtain the temperature of aggregation, *T*_agg_; (**B**) table of the average *T*_agg_ values of purified hAQP1 in the presence of hit fragments at increasing concentrations. Each value is the average of three independent measurements. These values are plotted as a histogram (**right**), where colors represent drug concentrations shown in Figure 8A.

Next, we examined whether these seven hits stabilize hAQP1 in a dose-dependent manner. In this secondary assay, the protein was incubated with increasing concentration of each fragment prior to the protein thermostability assay. For five fragments, the increase in thermal stability of the hAQP1 protein was clearly dependent on fragment concentration (Figure 8). These results confirmed the validity of these fragments as true binders to hAQP1. The results of the other two fragments, MO08607 and SB01744, were less consistent and are not shown. In the case of MO08607, the stabilization of hAQP1 was much better than in the initial screening where *T*_agg_ was 59.0 ± 0.3 °C at 1 mM (Figure 7). Here, *T*_agg_ of hAQP1 was 61.8 ± 0.6 °C in presence of just 50 μM of the compound, and increased to 66.3 ± 0.1 °C and 86.7 ± 7.2 °C in the presence of 400 and 2000 µM. Therefore, although showing inconsistent results, MO08607 can be considered a true binder. For SB01744, the *T*_agg_ of hAQP1 at different concentrations of the fragment could not be interpreted due to inconsistencies in the results, suggesting that this fragment is a false positive, or that its interaction with hAQP1 is non-specific.

As indicated in the introduction, the binders found should not necessarily show inhibition. Nevertheless, we performed stopped-flow assays to test the effects of the seven fragments in Figure 6 on hAQP1 water permeability using human red blood cells. Based on the values of the rate constants of the scattering increase, none of these fragments produced a significant reduction in water permeability when tested at 1 mM (not shown). Several factors may account for this. For example, binding does not occlude the pore as it may take place in extramembrane regions, or far from the lumen of the pore. In addition, hAQP1 in red blood cells is highly glycosylated compared to the hAQP1 purified from insect cells, and this may affect binding affinity. Another factor is the orientation of the protein in the membrane, as in theory only one side of the protein (extracellular) is exposed to the drug in RBC assays, whereas both extramembrane domains are exposed when the protein is solubilized in micelles. Another factor, also noted by Seeliger *et al.* [76], is the high density of AQP1 at the membrane of red blood cells. However, there was a large amplitude reduction in the traces for compounds CC38113 and CC46109, both highlighted in Figure 6A. It is interesting that we found previously a drug-like compound, NSC658139, that led to a strong reduction in overall amplitude [97]. This compound did not reduce the rate constant in the RBC assays but stabilized hAQP1 by as much as 13 °C at 30 µM. We are at present unable to explain this behavior or relate it to inhibition of hAQP1.

## 3. Discussion

To our knowledge, the data reported herein is the first fragment-based drug discovery approach to identify binders to an aquaporin using SPR and thermal shift assays. The use of these biophysical techniques that can detect weak intermolecular interactions is justified because the fragments screened in this work are expected to be weak binders due to their small size, and are unlikely to show inhibitory activity when tested in biological assays. Indeed, the affinity of the top 10 fragment binders identified using SPR was in the millimolar to high micromolar range.

In the SPR screening, more than 100 fragments (out of 1500) achieved surface occupancy above the baseline values. This apparent hit rate is much higher than that observed in conventional HTS screenings of drug-sized compounds because the complexity of the drugs and the probability of complementarity with the target are inversely correlated [105]. The second method measured protein thermostability under buffer conditions identical to those used in the SPR assay. Out of the 66 top binders detected in the SPR assay, seven were found to also increase the thermal stability of hAQP1, where most presented a dose-dependent behavior. This result suggests that good binders to hAQP1 may not necessarily stabilize the protein, at least when tested in these conditions. Conversely, fragments were found that produced good dose-response data in thermal shift but did not have a good dose-response behavior in SPR, despite appearing as binders in the primary SPR screen (see summarized results in Table 1).

Overall, the fragments selected from the two independent studies are anticipated to serve as good starting materials for the future design of more complex molecules that can become clinical candidates for hAQP1 inhibition. The next step will be to map the binding sites of these fragments on the hAQP1 protein surface using biophysical or structural techniques. High-resolution X-ray crystallography may be difficult for these low affinity compounds and may lead to low occupancy and low electron density. The binding epitope of the drug can also be studied by solution NMR, e.g., STD (Saturation Transfer Difference) experiments, which rely on the transfer of saturation from protein to ligand [106,107]. Direct protein resonance perturbation using labeled proteins is more difficult due to the large complex formed by tetrameric AQP and the detergent micelles. Finally, target perturbation can be tested using solid state NMR in lipid bilayers, but resonance assignments of hAQP1 are not yet available.

In principle, a fragment binding close to its pore could be developed into a more complex molecule that could physically occlude the channel. Possible chemical modifications include the linkage of two or more fragments or the customization of functional groups to build lead-like compounds. It is these lead-like compounds that are expected to be active in biological assays and can thus be tested using aquaporin functional assays to assess their inhibitory effects.

We propose the use of fragments as building blocks of aquaporin modulators. While there is still a long way to go before the hits from fragment screenings are able to deliver potent modulators of aquaporins, this strategy should be further developed as an alternative approach to the current saturation in the screening of drug-sized molecules for good therapeutic candidates against aquaporins. Specifically, AQP1 deletion directly causes reduced vascularity and hypoxia, which increases VEGF expression [55], suggesting that simultaneous inhibition of AQP1 and VEGF pathways could have an improved effect. In addition, anti-AQP1 therapies should not show adverse effects on humans because “Colton-null” individuals (AQP1 null) do not display significant clinical symptoms [108,109]. Therefore, AQP1 water channel inhibition may be a novel approach to reduce tumor vessels.

## 4. Materials and Methods

### 4.1. Purification of hAQP1 from Insect Cells

The full-length hAQP1 cDNA sequence was cloned into the vector pFB-LIC-Bse downstream of an N-terminal 6His and TEV protease cleavage site coding sequences (6His-TEV-hAQP1). Insect *Spodoptera frugiperda* cells overexpressing the recombinant hAQP1 protein was obtained from the Protein Production Platform (NTU, Singapore). The cell pellet was resuspended in 5 mL/g lysis buffer (20 mM Tris-HCl pH 8, 300 mM NaCl, 2 mM β-mercaptoethanol (β-ME), 10% glycerol (*v*/*v*), benzonase (25 U/mL) (Novagen, Madison, WI, USA), complete protease inhibitor cocktail and 2% *n*-Octyl-β-d-Glucopyranoside (OG, Anagrade, Affymetrix, Singapore)). Cell lysis was achieved with 10 min of sonication followed by passing through a microfluidizer (Microfluidics, Westwood, MA, USA) 5 times. The mixture was allowed to solubilize at 4 °C for 2 h, followed by centrifugation at 40,000× *g* for 30 min at 4 °C. The supernatant was loaded onto Ni-NTA column (packed with Bio-Rad Profinity™ IMAC resin, (Bio-Rad, Hercules, CA, USA) pre-equilibrated with lysis buffer. After overnight binding at 4 °C, the resin was washed with 30 resin volume of washing buffer (20 mM Tris-HCl pH 8, 300 mM NaCl, 25 mM imidazole, 2 mM β-ME and 10% glycerol). The final two resin volume wash was performed using washing buffer containing 50 mM imidazole and 1% OG (*w*/*v*) to remove additional contaminants. Recombinant hAQP1 protein was eluted with at least 5 resin volumes of elution buffer (20 mM Tris-HCl pH 8, 300 mM NaCl, 300 mM imidazole, 2 mM β-ME, 10% glycerol and 1% OG). Elution fractions were pooled and concentrated before gel filtration chromatography using HiLoad 16/600 Superdex 200 prep grade column (GE Healthcare, Uppsala, Sweden) at 4 °C. The protein was eluted with 20 mM Tris-HCl pH 8, 300 mM NaCl and 1% OG. The fraction size per elution is 2 mL, collected at a flow rate of 1 mL/min. *E. coli* AQPZ was prepared as indicated previously [101].

### 4.2. Gel Electrophoresis

The protein concentration of samples was measured using the NanoDrop™ 1000 spectrophotometer (Thermo Scientific, Waltham, MA, USA). SDS-PAGE gels were run at 200 V for 50 min, using TGS (25 mM Tris, 192 mM glycine, 0.1% SDS, pH 8.3) running buffer. The gels were stained with Coomassie blue (Bio-Rad, Hercules, CA, USA) and destained using 30% methanol and 10% acetic acid for visualization of protein bands. Protein standards were purchased from GE Healthcare (Vendevagen, Sweden).

Blue-native polyacrylamide gel electrophoresis, BN-PAGE, was performed as previously described [101]. Briefly, purified protein was incubated in sample buffer containing 750 mM aminocaproic acid, 50 mM Bis-Tris HCl pH 7.0, 0.5 mM EDTA and 100 mM OG (4 times its critical micellar concentration, CMC, of 25 mM). Coomassie brilliant blue was added to the sample to a concentration of 0.35% (*w*/*v*) immediately before gel loading. Samples were loaded into a pre-cast NativePAGE™ Novex™ 4%–16% Bis-Tris protein gel (Invitrogen, Carlsbad, CA, USA), with an inner blue cathode buffer (15 mM Bis-Tris HCl, 50 mM Tricine, and 0.02% Coomassie blue, pH 7.0) and an outer anode buffer (50 mM Bis-Tris HCl pH 7.0), and separated at a constant 150 V for approximately 70 min at 4 °C before replacing the blue cathode buffer with colourless cathode buffer (15 mM Bis-Tris HCl and 50 mM Tricine, pH 7.0), and allowed to run at 250 V till the dye front reached the edge of the gel. The NativeMark™ (Invitrogen) acted as the protein standard. The gels were stained with Coomassie blue.

### 4.3. Reconstitution of Purified Aquaporin into Liposomes.

Liposomes used in this work were prepared using film rehydration [110]. In this procedure, a thin lipid film of *Escherichia coli* lipids is formed by drying under a nitrogen stream lipid extract powder (Avanti Polar Lipids Inc., Alabaster, AL, USA) in chloroform. After at least 2 h in a vacuum desiccator, purified aquaporin protein in detergent was added and supplemented with reconstitution buffer (20 mM Tris–HCl pH 8.0) to obtain a protein-to-lipid molar ratio of 1:400. A volume of 1 mL of this reconstitution mixture was incubated for 1 h at room temperature. Uniform sized liposomes were obtained by extrusion through a 400 nm pore-size polycarbonate membrane using an Avestin extruder. Dynamic light scattering (90 Plus Particle Size Analyzer, Brookhaven Instruments, Holtsville, NY, USA) was used to measure the diameter of the AQP proteoliposomes. A liposome control was prepared in the same way without addition of protein.

### 4.4. Stopped-Flow Water Permeability Assay

The water permeability of proteoliposomes reconstituted with purified aquaporin was determined in a SX20 stopped-flow spectrometer (Applied Photophysics, Leatherhead, UK) with a dead time of 1.1 ms. Water permeability was assayed by rapidly mixing of a suspension of proteoliposomes with PBS containing 500 mM sucrose to establish a 250 mM inwardly directed osmotic gradient. The time course of 90° scattered light intensity at 500 nm was used to measure the kinetics of liposome shrinkage. Experiments were carried out at 23 °C. The solution osmolarities were measured using a vapor pressure osmometer (Wescor, Logan, UT, USA). Each experimental trace represents the average of five to ten replicates. The measured average diameter of control liposomes and hAQP1-proteoliposomes were 368 and 262 nm, respectively.

### 4.5. Light Scattering Data Analysis

Data was acquired by the Pro-Data SX software and analyzed by Pro-Data Viewer software (Applied Photophysics), where the measured light scattering intensity signals were fitted to an exponential function to obtain the rate constants. Dose-response curves were fitted using OriginPro 8.5 software (OriginLab, Northampton, MA, USA) to a sigmoidal function. The osmotic water permeability coefficients (*P_f_*) were calculated from the light scattering time course [102].

### 4.6. Surface Plasmon Resonance (SPR)-Protein Immobilization

Surface Plasmon Resonance measurements were performed using Biacore T-200 (GE Healthcare, Uppsala, Sweden) using the running buffer PBS pH 7.4 supplemented with 1% OG and 5% dimethylsulfoxide (DMSO) at 25 °C. Purified recombinant hAQP1 was directly immobilized onto Biacore Sensor Chip CM5 Research Grade (GE Healthcare, Little Chalfont, UK) using standard amine-coupling chemistry at a flow rate of 5 µL/min. Carboxyl derivatives on CM5 dextran surface were activated with a 10-min 1:1 mixture of 0.2 M *N*-ethyl-*N*′-(3-(diethylamino)propyl)carbodiimide (EDC) and 50 mM *N*-hydroxysuccinimide (NHS). The aquaporins were diluted in 10 mM sodium acetate, pH 5.0 and injected across the surface separately. Addition of 0.5 M ethanolamine–HCl at pH 8.5 for 10 min was used to block unreacted activated carboxyl derivates.

### 4.7. Fragment Screening Using SPR

The Maybridge Ro3 Diversity Fragment Library (Thermo Fisher Scientific, Waltham, MA, USA) consisting of 1500 fragments was used for screening. These fragments have molecular weight ranging from 82.1 to 295.3 Da, with an experimental guarantee of solubility of ≥1 mM in aqueous phosphate buffer and ≥200 mM in DMSO. They satisfy the “rule of three” criteria, *i.e.*, a molecular weight of ≤300 Da, a cLogP of ≤3.0, ≤3 H-bond acceptors or H-bond donors, ≤3 rotatable bonds, and a polar surface area of ≤60 Å^2^. Lyophilized powder of each fragments were pre-dissolved in 100% DMSO prior to the screening campaign. A volume of 10 µL of a 4 mM stock solution of each fragment in 100% DMSO was added to 190 µL of a buffer composed of 1.06 × PBS + 1.06% OG so as to obtain a final fragment concentration of 200 µM in PBS + 1% OG + 5% DMSO in a volume of 200 µL. We note that although a higher screening concentration in the mM range would in principle result in higher protein surface occupancy, allowing the detection of weaker interactions, this may also increase the probability of non-specific binding and/or promiscuously binding colloidal aggregates. In addition, the presence of salt and detergent in the buffer helps to stabilize the hAQP1 and also reduces the chances of non-specific binding. The DMSO solvent has a high refractive index and can be a major cause of false positives and plate-to-plate baseline variations. Therefore, it was important to accurately match its concentration in the sample buffer and the running buffer throughout the screening to minimize data inconsistencies during analysis.

The fragments were injected across a reference channel and hAQP1 channel for 2 min at flow rate of 30 µL/min, and then allowed to dissociate in running buffer for another min. Each fragment was injected in duplicates. DMSO calibration was performed for DMSO correction. Reproducibility was monitored with duplicate injections of each sample. No regeneration step was necessary as all fragments were dissociated. Previously validated positive controls, NSC226080 and NSC624169 (not shown), were included over the course of screening to monitor the surface protein activity.

Fragments that showed good binding response and reproducibility were further tested in a dose-dependent assay. These fragments, together with the positive controls, were diluted into 25, 50, 100 and 200 µM and tested over the same surfaces under the same conditions. DMSO calibration was performed for DMSO correction. Non-stoichiometric binders (potential fragments that aggregate, precipitate, binding non-specifically, *etc*.) were excluded. Good binders were assayed in a broader concentration range from 6.25 to 800 µM in 2-fold dilutions under the same conditions.

### 4.8. Data Processing and Curve Fitting

Raw sensorgrams were corrected for DMSO and double referenced (subtraction of the reference flow cell followed by subtraction of the closest buffer blank), which responses were corrected with a reference flow cell and a blank buffer injections. Corrected sensorgrams were *y*-axis and *x*-axis corrected at the point of fragment injection. Responses for the varied concentrations for a fragment were overlaid. Resultant equilibrium responses during injection phase were plotted against the log of their respective concentrations and fit to a steady-state model to obtain their affinities.

### 4.9. Thermal Aggregation Shift Assay

Temperature-dependent protein aggregation was measured with static light scattering (Stargazer-384, Harbinger Biotech, Markham, ON, Canada) as previously described [98] with modifications. Briefly, purified hAQP1 in PBS pH 7.4 and 1% OG at a concentration of 0.2 mg/mL, incubated with test compound or fragment at the appropriate concentration at room temperature for 15 min, and aliquoted in a clear-bottom 384-well plate (Nunc, Roskilde, Denmark) to a volume of 45 µL in triplicate. The final concentration of DMSO was kept at 5%. Mineral oil (45 µL) covered the sample to prevent evaporation. The sample was heated from 25 to 80 °C at a rate of 1 °C per minute. Incident light was shone on the protein drop from beneath at a 30° angle, and a CCD camera was used to measure the scattered light intensity every 30 s to monitor protein aggregation. Data collated was analyzed using the Bioactive software (Harbinger Biotech). Intensities were plotted as a function of temperature and fitted to the Boltzmann equation by non-linear regression. The point of inflection of the fitted curve defined the temperature of aggregation (*T*_agg_).

## Figures and Tables

**Table 1:** Summary of the main fragments identified in this work. Binding and stability data corresponding to 14 compounds (Figure 4 and Figure 6B). Note that, for AC15153, thermal shift data could not be obtained because the protein did not aggregate within the range of temperatures tested. *K*_D_ values for the last four compounds could not be obtained due to the bad quality of the sensorgrams performed at increasing concentrations. However, they were clearly identified as binders in the primary screening performed at 200 μM (Figure 3). Highlighted are the three compounds with good dose-responses in both SPR and positive (+) Thermal Shift experiments. Values of *T*_agg_ correspond to protein incubated in 1 mM of each fragment; errors are only given for positive thermal shifts. n.a., not applicable.

Maybridge Code	Structure	SPR (*K*_D_)	*T*_agg_ (°C)	Δ*T*_agg_ (+/−)
AC42399	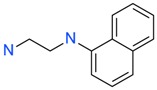	546 µM	48.0	−
MO00656	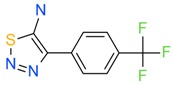	865 µM	51.8	−
CC53013	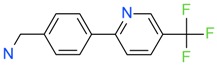	1.31 mM	48.6	−
CC38113	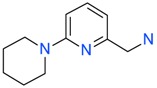	62 µM	63.3 ± 0.1	+
AC15153	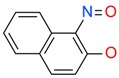	337 µM	n.a.	n.a.
SB00353	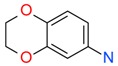	902 µM	51.1	−
MO08607	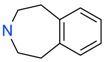	962 µM	59.0 ± 0.3	+
CC46109	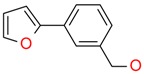	982 µM	103.4 ± 4.6	+
CC40813	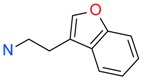	133 µM	50.3	−
AC34875	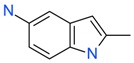	225 µM	50.9	−
CC47846	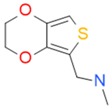	n.a.	70.0 ± 1.2	+
CC55714	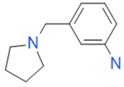	n.a.	63.6 ± 0.4	+
AC36708	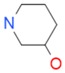	n.a.	62.9 ± 0.4	+
SB01744	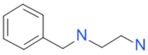	n.a.	58.7 ± 0.2	+

## Abbreviations

AQP	Aquaporin
DSLS	Differential Static Light Scattering
FBDD	Fragment-based Drug Discovery
HTS	High-throughput Screening
SPR	Surface Plasmon Resonance
TM	Transmembrane
